# Documentation of Phytotoxic Compounds Existing in *Parthenium hysterophorus* L. Leaf and Their Phytotoxicity on *Eleusine indica* (L.) Gaertn. and *Digitaria sanguinalis* (L.) Scop

**DOI:** 10.3390/toxins14080561

**Published:** 2022-08-18

**Authors:** HM Khairul Bashar, Abdul Shukor Juraimi, Muhammad Saiful Ahmad-Hamdani, Md Kamal Uddin, Norhayu Asib, Md. Parvez Anwar, SM Rezaul Karim, Ferdoushi Rahaman, Mohammad Amdadul Haque, Akbar Hossain

**Affiliations:** 1 Department of Crop Science, Faculty of Agriculture, University Putra Malaysia, Serdang 43400, Selangor, Malaysia; 2 Bangladesh Agricultural Research Institute (BARI), Gazipur 1701, Bangladesh; 3 Department of Land Management, University Putra Malaysia, Serdang 43400, Selangor, Malaysia; 4 Department of Plant Protection, Faculty of Agriculture, University Putra Malaysia, Serdang 43400, Selangor, Malaysia; 5 Department of Agronomy, Faculty of Agriculture, Bangladesh Agricultural University, Mymensingh 2202, Bangladesh; 6 Department of Agronomy, Bangladesh Wheat and Maize Research Institute, Dinajpur 5200, Bangladesh

**Keywords:** phytotoxins documentation, allelochemicals, caffeic acid, phytotoxicity, bioherbicides

## Abstract

The utilization of the invasive weed, *Parthenium hysterophorus* L. for producing value-added products is novel research for sustaining our environment. Therefore, the current study aims to document the phytotoxic compounds contained in the leaf of parthenium and to examine the phytotoxic effects of all those phytochemicals on the seed sprouting and growth of Crabgrass *Digitaria sanguinalis* (L.) Scop. and Goosegrass *Eleusine indica* (L.) Gaertn. The phytotoxic substances of the methanol extract of the *P. hysterophorus* leaf were analyzed by LC-ESI-QTOF-MS=MS. From the LC-MS study, many compounds, such as terpenoids, flavonoids, amino acids, pseudo guaianolides, and carbohydrate and phenolic acids, were identified. Among them, seven potential phytotoxic compounds (i.e., caffeic acid, vanillic acid, ferulic acid, chlorogenic acid, quinic acid, anisic acid, and parthenin) were documented, those are responsible for plant growth inhibition. The concentration needed to reach 50% growth inhibition in respect to germination (EC_g50_), root length (EC_r50_), and shoot length (EC_s50_) was estimated and the severity of phytotoxicity of the biochemicals was determined by the pooled values (rank value) of three inhibition parameters. The highest growth inhibition was demarcated by caffeic acid, which was confirmed and indicated by cluster analysis and principal component analysis (PCA). In the case of *D. sanguinalis*, the germination was reduced by 60.02%, root length was reduced by 76.49%, and shoot length was reduced by 71.14% when the chemical was applied at 800 μM concentration, but in the case of *E. indica*, 100% reduction of seed germination, root length, and shoot length reduction occurred at the same concentration. The lowest rank value was observed from caffeic acids in both *E. indica* (rank value 684.7) and *D. sanguinalis* (909.5) caused by parthenin. It means that caffeic acid showed the highest phytotoxicity. As a result, there is a significant chance that the parthenium weed will be used to create bioherbicides in the future.

## 1. Introduction

Crabgrass *Digitaria sanguinalis* (L.) Scop. and Goosegrass *Eleusine indica* (L.) Gaertn. are seasonal C_4_ plants [1] as well as tropical annual grass weeds that can be found in Africa, Asia, South America, and parts of North America, often causing problems in the production of highland crops. These weeds are one of the five most important destructive weeds in the world, hurting the yields of 46 different crop species in more than 60 countries [2,3]. It can withstand a wide range of salt concentrations, pH, and water stresses. Moreover, the seeds of goosegrass demonstrated a 79% viability at a depth of 20 cm after being buried for two years [4].

Agriculture faces a difficult problem when trying to manage weeds in crop fields. Because of their greater effectiveness, lower cost, and quicker payback, chemical herbicides are primarily favored by farmers to manage weeds. Another important issue for reliance in some countries is the transfer of labor away from agriculture to other industries or nations for jobs [5]. The impacts of climate change and health concerns are rising day by day due to the excessive use of synthetic herbicides, and we need effective alternatives to solve the weed management problem. Additionally, the primary requirements for the development of new selective herbicides are the ability to control the target plants at extremely low dosages that not harmful to the non-target organisms and to meet strict toxicological and environmental regulations. Understanding the mechanisms of herbicides’ selectivity would provide crucial knowledge for the development of novel herbicides [6].

The weed *Parthenium hysterophorus* L. is an invasive annual herbaceous weed which has global significance. Allelopathic chemicals can be released by this weed into the environment to suppress nearby competing plants. This weed causes allergic respiratory problems, contact dermatitis in human, cattle mutagenicity, and is a threat to crop production due to its potent allelopathic effects [7]. The management of this invasive and recalcitrant weed is an important issue in parthenium-infested countries, including Malaysia, through crop rotation, intercropping, cover cropping as living or dead mulches, green manuring, and use of allelochemical-based bioherbicides [8,9]. The utilization of this weed for extracting phytotoxic chemicals might be an option for parthenium management. So, the identification and separation of the allelopathic compounds from *P. hysterophorus* could be a technique for creating a bioherbicide. Terpenoids, steroids, phenols, coumarins, flavonoids, tannins, alkaloids, and cyanogenic glycosides, as well as their breakdown products, have been related to the allelopathic property of parthenium plants [10]. In terms of phytotoxicity, phenolic compounds have been the subject of the greatest investigation among these substances. These compounds are biologically active in suppressing weed seed germination and seedling growth [11]. The primary allelochemicals in parthenium were discovered in the phenolic compounds to be *p*-coumaric, *p*-hydroxybenzoic, ferulic acid, and vanillic acid [12]. At modest concentrations, the treatments with parthenin were also found to considerably delay germination but boost root growth [13]. The use of herbicides has expanded due to their budget-friendly solution to and labor-intensive technique of weed management. However, herbicide resistance in weeds has become more likely as a result of an over-reliance on them. For example, a good number of populations of crabgrass and goosegrass have evolved resistance to a variety of herbicides [14,15]. In the eight states of Malaysia, this grass has developed resistance to glyphosate, fluazifop, paraquat, and glufosinate [16]. Therefore, we need an alternative to chemical herbicides to solve the problems of weed resistance against herbicides. Bioherbicides are herbicides comprising phytotoxins, pathogens, and other microbes used as biological weed control [17]. Additionally, phytochemicals can be used as bioherbicides to boost crop output by biologically controlling weeds through allelopathy. By reducing the risk of mutagenic, genotoxic, and cytotoxic effects, these chemicals may also benefit human health [18]. Phytotoxicity, which can occur spontaneously or as a result of utilizing phytochemicals as bioherbicides, is one of the main effects of allelopathy [19]. Bioherbicides disintegrate quickly and do not leave residues in the soil after crops are harvested since they are based on natural chemicals and have short half-lives and process few halogen groups [20]. As a result, it is possible to control weeds using secondary metabolites derived from plants or other natural sources, helping to safeguard both people and the environment. On the other hand, bioassays are typically created to examine a plant species’ potential allelopathic effects. Because of the influence of several environmental circumstances, a plant that exhibits severe phytotoxicity toward the target plant species in laboratory conditions may not exhibit the same level of toxicity in the field context [21].

Some previous reports reveal the herbicidal potential of *P. hysterophorus* extracts in different plant species. As per our initial screening trials, parthenium leaf extracts have been examined to be a potential source of different allelochemicals with herbicidal and phytotoxic effects. However, inadequate evidence is available on the phytotoxicity of specific bioactive compounds which are identified in *P. hysterophorus* on the growth and development of crabgrass and goosegrass, which are the major weeds of rice and many of the field crops. Therefore, the main objective of this study is to evaluate the phytotoxicity of seven identified compounds from parthenium on the germination and growth of these weeds. The identification of its phytotoxic compounds was analyzed by using LC-ESI-QTOF-MS = MS (liquid chromatograph electrospray ionization quadrupole time of flight mass spectrometry).

## 2. Results

### 2.1. Identified Compounds from P. hysterophorus Leaf Methanol Extract through LC-MS Analysis

The identified compounds from *P. hysterophorus* leaf methanol extract through LC-MS analysis and their relative proportions of *P. hysterophorus* leaf with methanolic extract from positive and negative polarity analyses are listed in Table 1 and Table 2. From positive [M-H]^+^ polarity analysis, 33 compounds were identified from the leaf between retention time of 0.742 to 12.466 with *m*/*z* ratio of 112.1123 to 310.3452 and molecules mass of 94.0784 to 308.1963 (Table 1 and Table 2). With the concentration of 50 g L^−1^, there were three known toxic compounds detected from the leaf extract. On the other hand, negative (M-H^−^) polarity analysis detected 148 compounds between the retention time of 0.666 to 15.661 with the *m*/*z* ratio of 121.02988 to 1089.5495 and the mass molecules of 112.02781 to 2181.11278.

### 2.2. Documentation of Phytotoxic Compounds from P. hysterophorus Leaf Methanol Extract through LC–MS Analysis

The LC–MS analyses of *P. hysterophorus* leaf methanol extract revealed the presence of many compounds, such as terpenoids, flavonoids, amino acids, pseudo guaianolides, carbohydrates, and phenolic acids. Among them, phenolic acids are responsible for plant growth inhibition. The list of proposed phytotoxic compounds (caffeic acid, ferulic acid, vanillic acid, quinic acid, parthenin, chlorogenic acid, and *p* anisic acid) with their retention time, molecular formula, polarity, and mass fragment (*m*/*z*) is presented in Table 3.

**Figure 1:**
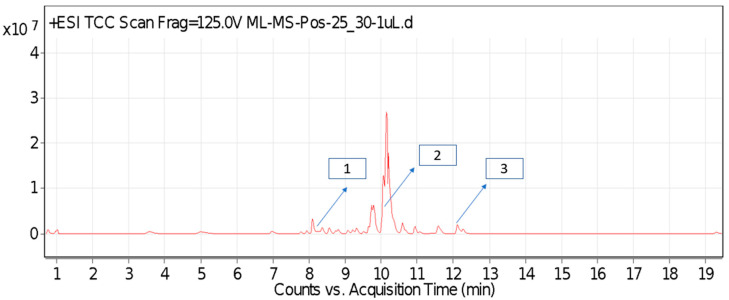
LC-MS chromatograms phytotoxic compounds on *P. hysterophorus* leaf methanolic extract positive ion mode (1. Chlorogenic acid, 2. Parthenin and 3. Quinic acid).

**Figure 2:**
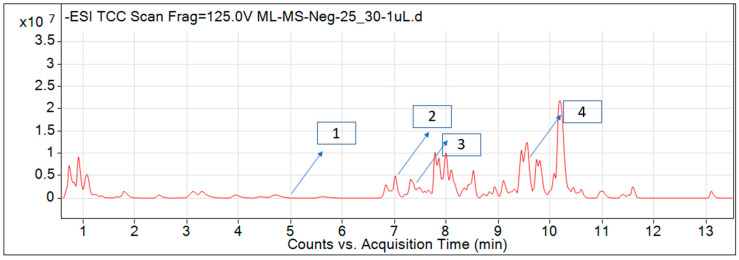
LC–MS chromatograms phytotoxic compounds on *P. hysterophorus* leaf methanolic extract positive ion mode (1. *p*-Anisic acid, 2. Caffeic acid, 3. Vanillic acid, and 4. Ferulic acid).

For most of the compounds, [M-H]^+^ and [M-H]^−^ ions were observed. The total ion current chromatography in positive and negative ESI mode is shown in Figure 1 and Figure 2. Quinic acid, parthenin and chlorogenic acid were identified by positive ionization mode at 12.116, 10.004, and 8.09 min, with 181.12, 263.1267, and 300.183 *m*/*z*, respectively. Another four phenolics, namely caffeic acid, ferulic acid, vanillic acid and *p*-anisic acid, were documented from negative polarity analysis at 7.183, 9.84, 7.367, and 5.121 min with *m*/*z* 341.0894, 193.05129, 153.01983, and 151.04047.

*P. hysterophorus* leaf extracts have a range of chemical compounds. Among them, phenolic compounds cause dermatitis, autotoxicity, and the suppression of other plants. There was a correlation between the quantity and kind of compounds detected in each plant and herbicidal activity.

### 2.3. Allelopathic Effects of the Phytochemicals on D. sanguinalis and E. indica

Significant killing effects of the chemicals on the test weed species were observed. The chemicals and their mixtures produced varying degrees of inhibitory effects on the germination, root growth, and hypocotyl elongation of *D. sanguinalis* and *E. indica*. The doses required for a 50% growth inhibition (EC_50_) of the weeds, as indicated by EC_g50_ (germination), EC_r50_ (root), and EC_s50_ (shoot) growth, were computed and found different from the control.

#### 2.3.1. Effects on Germination and Early Growth of *D. sanguinalis*

In the concentration–response bioassay, the inhibitory magnitude was increased for all compounds by increasing the concentration of chemicals from 100 μM to 1600 μM (Table 4). At the lowest concentrations (100 and 200 μM) of all tested compounds, less significant effect was found on the germination of *D. sanguinalis*, relative to the control except caffeic acid, which significantly suppressed the growth when applied with 200 μM; whereas for other chemicals, the germination percentage was significantly suppressed at rates higher than 400 μM. The germination of *D. sanguinalis* was severely decreased from 800 μM of chlorogenic acid, ferulic acid, parthenin, and vanillic acid. For quinic acid, anisic acid and a combination of compounds (mixture) inhibited growth when treated with 1600 μM. No germination was observed when treated with1600 μM of caffeic acid. Tested compounds did not exceed the doses to obtain EC_50_ employed in this study except caffeic acid, chlorogenic acid, and parthenin, which produced the highest growth inhibition at 100, 48, and 60%, respectively, and the lowest inhibition was caused by anisic acid (32%). Therefore, caffeic acid was the highest toxic in comparison to other chemicals in all the concentrations, followed by parthenin and chlorogenic acid.

Therefore, the phytochemicals have significant allelopathic effects on the root growth of the tested weeds. The root growth was significantly (*p* ≤ 0.05) reduced by caffeic acid, chlorogenic acid, quinic acid, parthenin, and a combination of their mixtures at all concentrations. Table 5 shows that caffeic acid, quinic acid, and parthenin were very toxic, reducing root development even at the lower doses (100 μM). An increase in the dose of these chemicals resulted in a higher degree of growth inhibition. The caffeic acid caused 76% inhibition at 800 μM, and from the 1600 μM concentration, no root was visible. Parthenin, quinic acid, chlorogenic acid, and a mixture of compounds, on the other hand, reduced the root growth by 60, 46, 47, and 47%, respectively, at a dose of 800 μM. The weakest inhibition (47%) was observed from ferulic acid even at the highest concentration.

A more or less similar pattern of effects on shoot length also occurred due to the treatments (Table 6). However, the shoot elongation of *D. sanguinalis* was not significantly decreased by a lower (400 μM) concentration of all compounds except caffeic acid, quinic acid, parthenin, and their mixtures. Vanillic acid, ferulic acid, and chlorogenic acid exhibited an adverse effect on the shoot elongation at 800 μM and beyond. On the other hand, only anisic acid exhibited a 43% inhibition at the highest concentration.

#### 2.3.2. Comparison between Phytochemicals in Their Effects on Growth Parameters

Table 7 shows some remarkable differences among the allelochemicals in terms of *D. sanguinalis* growth inhibition. The differences were apparent from the rank values of composites. Caffeic acid (R_e_ = 909.5) and parthenin (R_e_ = 2569.4) exposed higher inhibitory influences on the germination and development of *D. sanguinalis*; in other words, these compounds showed the most phytotoxic impact, which indicates that less concentration is needed to suppress this plant. While anisic acid (R_e_ = 14845.8), ferulic acid (R_e_ = 8878.8), and quinic acid (R_e_ = 8647.4) showed the weakest phytotoxicity compared to other chemicals. Consequently, it was apparent that the growth inhibitory effect of these compounds was the lowest. It means that anisic acid, ferulic acid, and quinic acid inhibit 50% of *D. sanguinalis* by more concentration than other tested compounds. According to Re value, the ranking of phytochemicals was caffeic acid < parthenin < chlorogenic acid < mixture < vanillic acid < quinic acid < ferulic acid < anisic acid. It can be mentioned here that the phytochemicals inhibited the growth of root length more than the growth of the shoot length and percent germination. The sum of EC_r50_ value for all compounds was 7811.2, whereas that of germination and shoot length were 32,597.9 and 54,700.8, respectively.

#### 2.3.3. Germination and Early Growth of *E. indica* Treated with Detected Allelochemicals

In the concentration–response bioassay, the inhibitory magnitude was increased for all compounds with increasing concentration from 100 μM to 1600 μM (Table 8). At the lowest concentrations (100 μM) of all tested compounds, less significant effect was found on the germination of *E. indica*, except by caffeic acid, chlorogenic acid, and the compound mixture. It significantly suppressed inhibition when applied at 200–1600 μM except for quinic acid and anisic acid. The germination of *E. indica* severely decreased from 800 μM of caffeic acid, chlorogenic acid, and parthenin.

On the other hand, vanillic acid, ferulic acid, quinic acid, anisic acid, and a combination of compounds inhibited the weed growth when treated with 1600 μM. However, no germination of weed seeds was observed when treated with 800 μM of caffeic acid. Tested compounds did not produce a significantly lower value of EC_50_ with the investigational doses used in this study except by caffeic acid, chlorogenic acid, and parthenin. The maximum growth inhibition of these compounds was observed at the rate of 100, 64, and 77%, respectively, and the lowest inhibition was found from anisic acid (15%). Therefore, it is obvious from the analysis that caffeic acid inhibited the most in all the concentrations, followed by parthenin and chlorogenic acid.

Identified allelochemicals have significant allelopathic effects on the root growth of the tested plant at varying doses (Table 9). All chemicals except vanillic acid significantly inhibited root elongation (*p* ≤ 0.05) at doses from 100 to 400 μM. However, at doses of more than 400 μM concentration, it suppressed the weed growth heavily. Table 9 shows that caffeic acid, quinic acid, and parthenin were strongly active, reducing root development even at the lowest concentration (100 μM). The caffeic acid produced 100% inhibition at an 800 μM concentration and above, while no root was visible, but 75 and 79% inhibition were observed from quinic acid and parthenin, respectively. The weakest phytotoxic effect (48%) on root development was noticed from chlorogenic acid at the highest concentration, while the rest of the compounds caused slightly more than 50% inhibition at the highest concentration.

A similar pattern of effect on shoot length was noticed as was on germination and root length (Table 10). The hypocotyl elongation of *E. indica* was not significantly decreased by a lower concentration (400 μM) of all compounds except caffeic acid, and vanillic acid. These compounds exhibited an adverse effect on the shoot elongation at the 800 μM and beyond. On the other hand, only anisic acid exhibited a 45% inhibition at the highest concentration.

#### 2.3.4. Comparison of Phytochemicals in Their Effects on Examined Initial Growth Parameters

Table 11 shows some remarkable differences among the identified allelochemicals in terms of the growth inhibition of *E. indica*. The differences were apparent from the rank values of composites. Caffeic acid (R_e_ = 684.7) and parthenin (R_e_ = 1637.66) showed the highest phytotoxicity on the germination and development of *E. indica*; in other words, these compounds showed the most phytotoxic impact, as indicated by the lower concentrations needed to suppress this plant. While anisic acid (R_e_ = 19553.25), ferulic acid (R_e_ = 7970.02), and a mixture (R_e_ = 5613.8) showed the weakest phytotoxicity compared to the others. The anisic acid, ferulic acid, and mixture of these compounds inhibit 50% of *E. indica* at a higher concentration than other tested compounds. The overall ranking, according to Re value, is caffeic acid < parthenin < vanillic acid < quinic acid < chlorogenic acid < mixture < ferulic acid < anisic acid. It is clear from the findings that the growth of root length is more affected by the chemicals than the growth of shoot length and percent germination. The sum of EC_r50_ values for all compounds was 9557.51, whereas the values for germination and shoot length were 27,149.61 and 48,847.3, respectively.

#### 2.3.5. Cluster Analysis and Assessment of Principal Component Analysis

**Figure 3:**
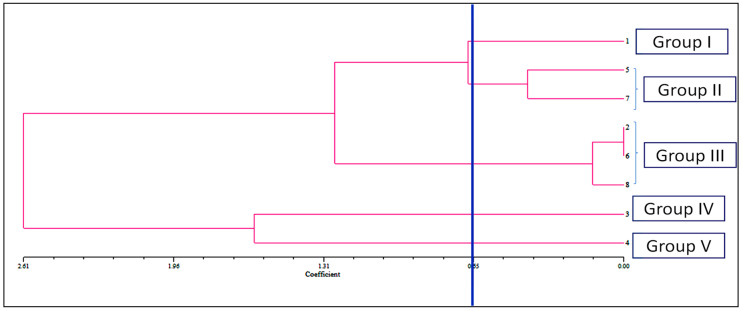
Dendrogram showing the mean EC_50_ values of seed sprouting, root, and hypocotyl length of *D. sanguinalis* and *E. indica* treated with the phytochemicals (1. caffeic acid, 2. vanillic acid, 3. ferulic acid, 4. chlorogenic acid, 5. quinic acid, 6. anisic acid, 7. Parthenin, and 8. Mixture) revealed by non-overlapping (SAHN) as produced by the UPGMA method.

The allelopathic activities of examined compounds and their combination in bioassay were clustered into four interpretable groups, according to the dendrogram (group I–V) as indicated. In the dendrogram, there was a coefficient cut-off at 0.65 for ease of interpretation (Figure 3). Group I consisted of caffeic acid, which was characterized by the most inhibitory effects and with low-rank values. Parthenin and quinic acid are in group II with stronger inhibitory effects; Group III is comprised of vanillic acid, anisic acid, and mixture; group IV consists of ferulic acid; and chlorogenic acid is in group V, which had moderate inhibitory effects. The compounds under groups IV and V demonstrated a relatively weak phytotoxic effect in comparison with other groups.

**Figure 4:**
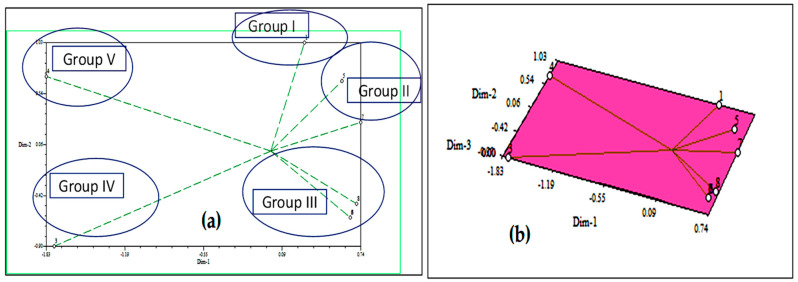
Based on Euclidian distance, principal component analysis (PCA)-2D graphical relationship between the discovered allelochemicals; (**a**) eigenvectors and (**b**) eigenvalues.

The effects of *D. sanguinalis* and *E. indica* were responsible for the majority of the differences observed in the cluster. The two-dimensional and three-dimensional (Figure 4) graphical elucidations confirmed that the maximum of the phytochemicals was discrete at low distances, the only two were discrete at long distances as represented by the eigenvector. The furthest accessions from the centroid were 3 and 4, whereas others were close to the centroid.

## 3. Discussion

The *P. hysterophorus* extracts contained a large number of chemicals that were discovered using phytochemical screening, some of which had previously been recognized as toxins in other studies [28,29,30,31,32,33]. Furthermore, a variable number of chemicals were also present in different plant parts of *P. hysterophorus*. The leaf has a stronger inhibitory impact since it contains more harmful chemicals than the other plant parts. The suppressive influence of extracts, according to Verdeguer et al. [34] is determined by the extract’s chemical makeup as well as the plant sections to which it is applied. These findings are consistent with those of Javaid and Anjum [35] and Verma et al. [36] who discovered that the main causes of the inhibition of plant growth are parthenin and other phenolic acids including caffeic acid, vanillic acid, anisic acid, chlorogenic acid, and para-hydroxybenzoic acid.

In this investigation, tested the phytotoxicity of all previously identified allelopathic compounds. The pure compound bioassay (chemicals purchased from the market) demonstrated that all of the examined compounds and their mixtures were physiologically dynamic and toxic, reducing seed germination and development in crabgrass and goosegrass. These results confirmed that the compounds found in *P. hysterophorus* are potential allelochemicals and that they are most likely responsible for *P. hysterophorus’* herbicidal behavior. Caffeic acid, chlorogenic acid, and parthenin were the most active of the compounds tested (Table 2 and Table 6). In fact, the plant’s allelochemicals have yet to be discovered.

Our results are also supported by the results of others [11,37,38,39,40,41,42] who discovered that caffeic acid, benzoic acid, *p*-anisic acid, chlorogenic acid, *trans*-ferulic acid, *trans*-cinnamic acid, and syringic acid had an allelopathic effect on the seed germination and early growth of *Phaseolus vulgaris, Phaseolus aureus, Arabidopsis thaliana, Echinochloa crus-galli, Lactuca sativa*, and *Sagittaria montevidensis*, respectively, despite clear dose–response differences.

According to Bajwa et al. [42] and Guo et al. [43] the extracts from allelopathic plant species produce much higher total phenolics than extracts from non-allelopathic plant species. The most vital and prevalent plant allelochemicals in the environment are phenolic derivatives [44]. Numerous papers have focused on the allelopathic and phytotoxic characteristics of phenolic and flavonoid chemicals [42,45]. Phenolic derivatives are an important class of allelopathic chemicals with a wide range of allelopathic actions. Regardless of dose, these components exhibited the most negative impact on seed germination and the development of barnyard grass [46]. Plant growth and development are inhibited by phenolic acids, which are one of the principal groups of metabolites implicated in allelopathic interactions in the soil atmosphere [47]. Amarowicz et al. [48] discovered that phenolics from the Jerusalem artichoke (*Helianthus tuberosus* L.) influenced lettuce development. According to Braga et al. [49], flavonoids inhibited the growth of standard target species (STS), such as *Lactuca sativa* (lettuce), *Lycopersicon esculentum* (tomato), and *Allium cepa* (Onion). Parthenin, chlorogenic acid, and ambrosian were also found to be favorably connected with germination inhibition and radicle elongation inhibition [42]. Caffeic acid, chlorogenic acid, ferulic acid, gallic acid, *p*-coumaric acid, 4-hydroxy-3-methoxybenzoic acid, m-coumaric acid, syringic acid, and vanillic acid were found as phytotoxins in parthenium, which cause allelopathic effects on crops [50]. Caffeic acid was shown to be the most effective inhibitor, as measured by thin-layer chromatography, melting point, infrared spectrum studies, and seedling emergence reduction [38,51].

*P. hysterophorus* extracts were found to have a higher inhibitory effect than individual compounds and even a mixture of all identified components [52]. The extracts’ stronger inhibitory effects could be owing to unique chemical combinations that work in an additive or synergistic manner. This shows that undiscovered extract components may have a synergistic effect on phytotoxic action, if not direct activity [53]. It can be speculated that in addition to the established phenolic and flavonoid components, unknown chemicals are responsible for the overall allelopathic impact of extracts. Mixtures of phenolic compounds were less suppressive as compared to the allelopathic activity of individual phenolic compounds (Table 2 and Table 6), which might be due to the fact that the allelopathic effect is regulated by concentration interactions, chemical combinations, and test species sensitivity because growth inhibition in mixes is lower than in individual component chemicals [54].

## 4. Conclusions

From the LC–MS analysis, many compounds, such as terpenoids, flavonoids, amino acids, pseudo guaianolides, and carbohydrate and phenolic acids, were identified from positive and negative polarity analysis. Among them, seven known phenolic derivatives were documented from the *P. hysterophorus* leaf methanol extract, which was responsible for plant growth inhibition. Seed germination and the development of crabgrass and goosegrass was reduced by all of the compounds, indicating that all combinations of all compounds were physiologically active. Caffeic acid and parthenin had the maximum phytotoxicity on crabgrass and goosegrass during germination and seedling development; indicating that a lower dosage is required to inhibit this plant. In comparison to the others, anisic acid, ferulic acid, and combination demonstrated the least phytotoxicity. This means that these chemicals need to inhibit to a greater extent than other chemicals on crabgrass and goosegrass germination and seedling growth to achieve the same effect. Overall, the ranking values were caffeic acid < parthenin < vanillic acid < quinic acid < chlorogenic acid < mixture < ferulic acid < anisic acid. Among these tested compounds, caffeic acid, chlorogenic acid, and parthenin were found to be the most active, and thus might be appropriate candidates for developing bioherbicides.

## 5. Materials and Methods

### 5.1. Site Description

The experimentation was conducted in the weed science laboratory of the Department of Crop Science, Faculty of Agriculture, Universiti Putra Malaysia (UPM), Serdang, Selangor, Malaysia. Liquid Chromatography–Mass Spectrophotometry (LC-MS) analysis was carried out at Monash Universiti, Malaysia.

### 5.2. Extract Preparation

Parthenium leaves were collected from the Ladang Infoternak farm in Sungai Siput, Perak, Malaysia. Plants leaf was collected randomly during the vegetative stage (15–20 days old plants), rinsed with tap water numerous times to remove dust particles, and air-dried for three weeks at room temperature (24–26 °C). In a laboratory blender, plant leaves were mashed into a fine powder and sieved through a 40-mesh sieve.

The extracts were made according to the procedure described by Ahn and Chung [55] and Aslani et al. [56]. An amount of 100 g leaf powder of parthenium was placed in a conical flask and allowed to soak in 1 L of 80% (*v*/*v*) methanol. The conical flask was wrapped in paraffin and shaken for 48 h at 24–26 °C room temperature in an orbital shaker at a 150 rpm agitation speed. To remove debris, cheesecloth in four layers were used to filter the mixtures. The supernatant was centrifuged for one hour at 3000 rpm in a centrifuge (5804/5804 R, Eppendorf, Germany). A single layer of Whatman No. 42 filter paper was used to filter the supernatant. A 0.2-mm Nalgene filter (Lincoln Park, NJ-based Becton Dickinson percent Labware) was used to filter the solutions one more time to exclude microbial development. Using a rotary evaporator (R 124, Buchi Rotary Evaporator, Germany), the solvents were evaporated from the extract to dryness (a thick mass of coagulated liquid) under vacuum at 40 °C and the sample was then collected. From a 100 g sample of *P. hysterophorus* powder, the average extracted sample was 17.56 g, which was estimated as per the following formula [57]:

[Extract weight (g)/powder weight (g)] *×* 100 = Extraction percentage (1)

All extracts were stored at 4 °C in the dark until use. For LC–MS analysis, 100% HPLC GRADE methanol (20 mL) was diluted with the crude sample (20 mg) and filtered through 15-mm, 0.2-μm syringe filters (Phenex, Non-sterile, Luer/Slip, LT Resources Malaysia).

### 5.3. Identification of Phytotoxic Compounds from P. hysterophorus Leaf Methanol Extract

The analysis of the phytochemical compounds of the methanol extracts was performed using LC–MS followed by Schimanski et al. [58]. LC–MS analysis was carried out using Agilent spectrometry equipped with a binary pump. The LC–MS was interfaced with the Agilent 1290 Infinity LC system coupled to Agilent 6520 accurate-mass Q-TOF mass spectrometer with a dual ESI source. Full-scan mode from *m*/*z* 50 to 500 was set with a source temperature of 125 °C. The column of Agilent zorbax eclipse XDB-C18, narrow-bore 2.1 × 150 mm, 3.5 microns (P/N: 930990-902) was used at the temperature of 30 °C for the analysis. A—0.1% formic acid in water—and B—0.1% formic acid in methanol—were used as solvents. Isocratic elution was used to supply solvents at a total flow rate of 0.1 mL minutes^−1^. MS spectra were collected in both positive and negative ion modes. The drying gas was 300 °C, with a 10 mL min-1 gas flow rate and a 45-psi nebulizing pressure. Before analysis, sample extraction was diluted with methanol and filtered through a 0.22 m nylon filter. The extracts were injected into the analytical column in 1 μL volume for analysis. The mass fragmentations were discovered using an Agilent mass hunter qualitative analysis B.07.00 (Metabolom-ics-2019.m) tool and a spectrum database for organic chemicals.

### 5.4. Experimental Treatments and Layout

The treatments consisted of seven biochemicals e.g., caffeic acid, vanillic acid, ferulic acid, chlorogenic acid, quinic acid, anisic acid, and parthenin at different concentrations of 0 (distilled water), 100, 200, 400, 800, and 1600 μM., and two weed species, crabgrass, and goosegrass. Completely randomized designs (CRD) with four replications were used to arrange the experimental units (Petri dishes).

### 5.5. Plant Materials and Compounds

These detected seven phytotoxic compounds were purchased from Bio-solutions Sdn Bhd, Kuala Lumpur, Malaysia. The source of all chemicals is Sigma-Aldrich (St. Louis, MO, USA). The seeds of two weed species, crabgrass and goosegrass, were collected from UPM agricultural field and then kept in a refrigerator for 15 days at 4 °C for further use.

### 5.6. Bioassay

Individual chemicals and their mixtures were tested for their inhibitory effects on the germination and early growth of the weed species. Six different concentrations of the chemicals were achieved by dissolving the appropriate amount of chemicals in distilled water, i.e., 1600, 800, 400, 200, 100, and 0 μM (control), which were then sonicated at 60 kHz for one hour at 30 °C in an ultrasonic bath. The precise process for making various chemical concentrations includes dissolving the right amount of powder based on their molecular weight, such as the molecular weight of caffeic acid, i.e., 180.16 g. Thus, 1 mol equals 180.16 g. Therefore, a 1 molar solution will result from diluting 1 liter of distilled water by 180.16 g caffeic acid. Consequently, 1600 moles = (1600 × 180.16) = 540,480 g. In this manner, 540.48 mg of powder is required to create a 1000 mL solution in distilled water [59].

Healthy and uniform weed seeds were gathered and soaked for 24 h in 0.2% potassium nitrate (KNO3), then rinsed with distilled water and incubated at room temperature (24–26 °C) until the radicle emerged by about 1 mm. Thirty uniform pre-germinated seeds were inserted in disposable plastic 9.0-cm-diameter Petri dishes with two sheets of Whatman No. 1 filter paper. After that, the filter paper on the Petri dishes was wetted and soaked with 5 mL of six different chemical solutions. In the same way, 5 mL of pure water was treated as a control. The Petri dishes were then incubated under fluorescent light (8500 lux) in a growth chamber at 30/20 °C (day/night) with a 12 h/12 h (day/night cycle). The relative humidity ranged from 30% to 50%. To facilitate gas exchange and avoid anaerobic conditions, the lids of the Petri dishes were not sealed.

### 5.7. Data Measurement

Seed germination was counted, and root and shoot lengths of the weed species were measured after 1 week of seed placement with a ruler. The radicle and hypocotyl length was measured using Image J software (https://imagej.nih.gov/ij/docs/guide/user-guide.pdf; accessed on 10 July 2022) [60]. The inhibitory effect of *P. hysterophorus* extracts on germination, radicle length, and hypocotyl length was computed following the equation [25]:

I = 100 (C − A)/C
(2)

where “I” is the percentage of inhibition, “C” is the control’s mean, and “A” is the treatment (extract) mean of germination, radicle length, and hypocotyl length.

To find discrete groupings of allelochemicals with similar phytotoxicity, the most common application of NTSYSpc 2.02e (Numerical Taxonomy and Multivariate Analysis System) was used to perform various types of agglomerative cluster analyses and to estimate some type of similarity or dissimilarity matrix to further define the level of sensitivity to chemical compounds among the plants under investigation [59,60].

Effective dosages capable of suppressing 50% of germination, root length, and shoot length were calculated using EC_g50_, EC_r50_, and EC_h50_, respectively. The EC_g50_, EC_r50_, and EC_h50_ values were calculated using Probit analysis based on the percent of root and shoot length inhibition, respectively. The following equation was used to create an index (Re) for each of the most active extracts and the most sensitive plants for each plant tested:

EC_g50n_ (germination) + EC_r50n_ (root) + EC_h50n_ (shoot) = Rank (R_e_)

where Re is the plant’s rank n and EC_g50n_, EC_r50n_, and EC_h50n_ are the amounts of plant extract n that inhibit 50% of germination, root length, and shoot length, respectively. The lowest Re value had the most active chemical and the most sensitive plants, while the highest Re value had the least inhibition effect on the chemicals.

### 5.8. Identified Compounds from P. hysterophorus Leaf Extract

The identified compounds and their relative proportions of the *P. hysterophorus* leaf with methanolic extract from positive and negative polarity analyses are listed in Table 1 and Table 2.

### 5.9. Details of the Phytotoxic Compounds

Details of the phytotoxic compounds (i.e., retention time, *m*/*z*, mass, polarity, synonyms, chemical formula and structure and biological activity with proper citations) of *P. hysterophorus* leaf with methanolic extracts through LC–MS analysis are available in Table 3.

### 5.10. Statistical Analysis

The data (germination percentage, root length, and shoot length) is transformed by the log transformation {log_10_ (x + 1)} system. The variance homogeneity was evaluated using Levene’s test. The data normality was analyzed using Shapiro–Wilk tests and after transformation, the data is assumed to be normally distributed. Two-way analysis of variance (ANOVA) was performed (two factors: concentrations and chemical compounds; fixed factor: weed species) using R-studio software to evaluate whether there was a significant difference between each treatment and the control, after that, the LSD test was used to separate the treatment and control means at 0.05 probability levels

## Figures and Tables

**Table 1:** Identified compounds in the methanol extract of leaf of *P. hysterophorus* from LC–MS positive polarity analysis.

Compounds	Retention Time	*m*/*z*	Mass	Group	References
1-Methyl-1,3-cyclohexadiene	0.742	112.1123	94.0784	Oils	[22]
Octylamine	5.022	130.1591	129.1518	Unknown	
3-(1-Pyrrolidinyl)-2-butanone	0.779	142.1227	141.1154	Unknown	
Quinic acid	12.116	181.12	180.1129	Phenolics	[22,23]
3,5-dimethyl-Phenol	9.084	197.1164	196.1091	Volatile oils	[24]
Flossonol	10.013	221.1164	220.1091	Unknown	
3,4,5-Trimethoxyphenyl acetate	3.583	227.0923	226.085	Unknown	
Undecenyl acetate	11.071	235.1675	212.1782	Unknown	
Lumichrome	9.802	243.0879	242.0807	Unknown	
D-Biotin	10.156	245.0963	244.089	Unknown	
L-beta-aspartyl-L-leucine	10.065	247.1293	246.1221	Amino acids	[22,25]
Histidylproline diketopiperazine	10.117	249.1346	248.1275	Amino acids	
4,5-Dihydrovomifoliol	10.356	249.1455	226.1563	Volatile oils	[24]
Carbamic Acid tert-butyl ester	9.522	249.1468	231.113	Unknown	
Grosshemin (Parthenin)	10.004	263.1267	262.1194	Terpenoids, Phenolics	[22,25,26]
Sudan Brown RR	9.658	280.1556	262.1217	Unknown	
16-hydroxy hexadecanoic acid	12.274	290.2681	272.2342	Amino acids	[25]
Artecanin	8.592	296.1484	278.1145	Terpenoids	[25]
Autumnolide	8.738	298.1637	280.1298	Unknown	
Hymenoflorin	9.323	298.1655	280.1316	Terpenoids	[25]
N-Histidyl-2-Aminonaphthalene	8.372	298.1658	280.132	Unknown	
EHNA	8.18	300.1794	277.1901	Unknown	
Artemisinin	9.229	300.1795	282.1456	Terpenoids	[25]
Dihydroartemisinin	8.24	302.1951	284.1604	Terpenoids	[25]
Ligulatin B	11.653	324.1801	306.1464	Terpenoids	[25]
Tetraneurin A	9.918	340.176	322.1421	Pseuguaianolids	[22]
Chlorogenic acid	8.09	300.183	282.1482	Phenolics	[22,26]
4-O-Demethyl-13-dihydroadriamycinone	8.555	403.1035	402.0961	Unknown	
Cynaroside A	7.774	462.2336	444.1996	Flavonoids	
Maltotriitol	10.154	507.1936	506.1861	Unknown	
Hexafluoro-25-hydroxycholecalciferol	10.109	531.2666	508.2779	Unknown	
p-benzamidophenyl ester	10.099	548.2992	547.292	Unknown	
7-Deacetoxy-7-Oxokhivorin	10.69	560.2848	542.2514	Unknown	

Note: *m*/*z* = mass number/charge number means mass-to-change ratio.

**Table 2:** Identified compounds in the methanol extract of leaf of *Parthenium hysterophorus* from LC–MS negative polarity analysis.

Compounds	Retention Time	*m*/*z*	Mass	Group	References
(-)-12-hydroxy-9,10-dihydrojasmonic acid	11.517	227.12948	228.13676	Volatile oils	[24]
^®^-3-^®^-3-Hydroxybutanoyloxy) butanoate	8.163	189.07773	190.08498	Unknown	
1,3,7-Trimethyluric acid	0.682	209.06864	210.07593	Unknown	
1,3,8-Trihydroxy-4-methyl-2,7-diprenylxanthone	6.831	393.1689	394.17594	Unknown	
16-bromo-9E-hexadecanoic acid	8.268	367.10596	332.13723	Amino acids	[27]
17-α, 1-Dihydroxy-11,20-dioxo-5-β-pregnan-3-α-yl-β-d-glucuronide	10.169	539.24843	540.24875	Unknown	
1 alpha,5alpha-Epidithio-17a-oxa-D-homoandrostan-3,17-dione	9.818	365.12623	366.13357	Unknown	
1-Methylhypoxanthine	0.725	149.04721	150.05456	Unknown	
2,2,4,4-Tetramethyl-6-(1-oxobutyl)-1,3,5-cyclohexanetrione	9.778	251.13009	252.13678	Unknown	
2,3-dimethyl-3-hydroxy-glutaric acid	3.108	175.06173	176.06896	Carbohydrate	[22]
2,3-dinor Thromboxane B1	10.89	343.21388	344.22105	Unknown	
2,4,6-Triethyl-1,3,5-oxadithiane	3.668	205.07267	206.08007	Unknown	
2,4-Diamino-6,7-dimethoxyquinazoline	7.794	219.08808	220.09536	Unknown	
3-(4-Hydroxy-3-methoxyphenyl)-1,2-propanediol 2-O-(galloyl-glucoside)	7.299	511.14783	512.15661	Carbohydrate	[22]
3,4-Dihydroxybenzoic acid (Vanillic acid)	7.367	153.01983	154.02711	Phenolics	[22,25,26]
3-Amino-3-(4-hydroxyphenyl) propanoate	0.998	180.06692	181.07393	Amino acids	
3-carboxy-4-methyl-5-propyl-2-furanpropanoic acid	8.307	239.09312	240.10037	Amino acids	
3H-1,2,4-Triazol-3-one, 5-ethyl-2,4-dihydro-2-(3-hydroxypropyl)-4-(2-phenoxyethyl)-	10.076	326.12684	291.15819	Unknown	
3-Hydroxy-3-methyl-2-oxo-Butyric acid	1.633	131.03516	132.04241	Oils	[22]
3-Hydroxylidocaine	9.798	285.13672	250.16761	Amino acids	
3-Methoxy-4-hydroxyphenylglycol glucuronide	4.138	359.09968	360.10747	Carbohydrate	[22]
3-propylmalic acid	3.918	175.06159	176.0688	Unknown	
4-(3-Methylbut-2-enyl)-L-tryptophan	10.178	307.12149	272.152	Amino acids	[27]
4,4′-Stilbenedicarboxamidine	10.027	263.13026	264.13744	Unknown	
4-Cyano-4-(3,4-dimethoxyphenyl)-5-methylhexylamine	10.128	311.15235	276.18326	Unknown	
4-Hydroxyphenylpyruvic acid	7.635	179.03577	180.04303	Amino acids	[27]
5,8,12-trihydroxy-9-octadecenoic acid	11.435	329.23494	330.24207	Amino acids	[27]
7-beta-D-Glucopyranosyloxybutylidenephthalide	9.821	365.12586	266.1332	Unknown	
Abruquinone C	10.18	375.10912	376.11641	Flavonoids	[25]
Absindiol	10.36	301.12166	266.15233	Terpenoids	[24]
AFMK	6.439	299.07923	264.10991	Unknown	
Ala Tyr Pro	9.747	384.1318	349.1631	Unknown	
alpha-Carboxy-delta-decalactone	10.168	213.11385	214.12108	Unknown	
Amlodipine	8.963	407.137	408.14433	Flavonoids	[25]
Apodine	8.371	401.12848	366.15935	Flavonoids	
Apuleidin	0.966	359.07565	360.08359	Flavonoids	
Asparagoside F	11.384	516.259	1034.5319	Flavonoids	
Austalide C	9.327	573.23642	574.24438	Flavonoids	[25]
Baccatin III	10.962	585.23644	586.24356	Unknown	
Benzocaine	1.797	164.07181	165.07903	Unknown	
Benzyl O-[arabinofuranosyl-(1->6)-glucoside]	9.634	401.14728	402.15456	Carbohydrate	[22]
beta-Snyderol	0.718	299.10119	300.10852	Unknown	
Caffeic acid	7.183	341.0894	342.09698	Phenolics	[22,25,26]
Cardiogenol C	8.864	259.12027	260.12766	Flavonoides	[25]
Carteolol	9.245	327.1469	292.17758	Unknown	
Cys Arg Asn	8.928	390.1574	391.16503	Amino acids	[27]
Cys Asp Trp	7.819	421.1202	422.12913	Amino acids	[27]
Delavirdine	9.697	491.16194	456.19328	Unknown	
Diethyl (2R,3R)-2-methyl-3-hydroxysuccinate	9.422	203.09338	204.10062	Unknown	
Dihydroartemisinin	7.893	283.15577	284.16289	Flavonoids	[25]
Diphenylcarbazide	10.381	241.10929	242.11671	Unknown	
Enoxacin	9.817	355.09854	320.12917	Flavonoids	
Ent-afzelechin-7-O-beta-D-glucopyranoside	9.752	435.1295	436.13611	Cabohydrate	
Eremopetasitenin B2	9.553	463.17991	464.18582	Terpenoids	
Ethotoin	4.401	203.08308	204.09031	Flavonoids	
Ethyl (S)-3-hydroxybutyrate glucoside	6.87	293.12519	294.13239	Carbohydrate	[22]
Ethyl 3-hydroxybutyrate	7.386	131.07191	132.07915	Unknown	
Ethyl Oxalacetate	3.317	187.06185	188.06916	Unknown	
Fenspiride	9.779	295.12102	260.15156	Unknown	
Ferulic acid	9.84	193.05129	194.05855	Phenolics	[22,25,26]
Florilenalin	10.259	299.10472	264.13681	Terpenoids	[25]
Fluvoxamine acid	4.695	353.0896	318.12038	Terpenoids	[25]
Formononetin 7-O-glucoside-6”-O-malonate	9.121	515.12161	516.12902	Unknown	[25]
Ganglioside GT1b (d18:1/22:1(13Z))	10.45	1089.5495	2181.1127	Unknown	[25]
Gingerol	13.113	293.1772	294.18435	Terpenoids	[25]
Gitonin	10.546	524.25681	1050.5274	Unknown	[25]
Gly Val	0.692	209.06916	174.09982	Unknown	[25]
Glycobismine A	8.04	601.23429	602.24125	Terpenoids	[25]
Granisetron metabolite 4 glucuronide	7.266	489.19925	490.20683	Terpenoids	
Guanosine	1.22	282.08553	283.09279	Unknown	
Hinokitiol glucoside	8.384	325.13062	326.13781	Carbohydrate	[24]
Imazethapyr	10.179	324.11121	289.14209	Unknown	
Isobavachalcone	7.011	323.12875	324.13481	Terpenoids	[25]
Isoetin 4′-glucuronide	8.936	477.06972	478.07703	Terpenoids	[25]
Isopropyl β-D-Thiogalacto Pyranoside	3.041	237.08105	238.08826	Unknown	
Isoxaben	9.746	367.14195	332.17282	Phenolics	[22,25,26]
LPA(18:2(9Z,12Z)/0:0)	9.989	469.21108	434.24192	Unknown	
Leukotriene F4	6.989	603.25077	568.28124	Unknown	
Levoglucosan	1.872	161.04586	162.05315	Unknown	
Licoagrone	10.094	370.13067	742.28883	Flavonoids	[25]
Maltopentaose	9.11	863.24564	828.2779	Unknown	
Manumycin A	10.612	585.23644	550.26916	Unknown	
Melleolide L	8.696	485.11475	450.14606	Unknown	
Mepiprazole	8.597	303.13857	304.14582	Unknown	
Methitural	1.936	287.09005	288.0975	Unknown	
Methyl ^®^-9-hydroxy-10-undecene-5,7-diynoate glucoside	9.062	367.14164	368.14881	Carbohydrate	[22]
Methyl 2-(4-isopropyl-4-methyl-5-oxo-2-imidazolin-2-yl)-p-toluate	6.102	323.11808	288.14871	Unknown	
Methyl 6-O-digalloyl-beta-D-glucopyranoside-	10.076	309.13704	310.14433	Unknown	
Methylthiomethyl 2-methylbutanethiolate	0.925	177.04192	178.0498	Unknown	
Mitoxantrone	7.792	479.17082	444.20125	Unknown	
Monodeallydihydroxyalmitrine	7.792	506.18981	471.22059	Unknown	
Metofluthrin	7.452	395.10599	360.13672	Terpenoids	[25]
N-Ac-Tyr-Val-Ala-Asp-CHO	6.791	491.21465	492.22186	Unknown	
Nomilinic acid 17-glucoside	7.361	747.2638	712.2946	Carbohydrate	[22]
Novobiocin	8.00	611.22246	612.22878	Flavonoids	
N-Benzoylaspartic acid	7.193	236.0572	237.06511	Unknown	
N-Carboxytocainide glucuronide	4.809	447.11614	412.14707	Terpenoids	
N-Feruloylglycine	7.739	250.07333	251.08065	Unknown	
N-Histidyl-2-Aminonaphthalene (βNA)	8.382	279.12482	280.13203	Unknown	
N-isovalerylglycine	4.074	158.08237	159.08961	Amino acids	
O-b-D-Gal-(1->3)-O-2-(acetylamino)-2-deoxy-D-Galactose	7.361	747.26473	748.27167	Carbohydrate	
Octadecanoic acid-1,2,2,2-tetrafluoro-1-(trifluoromethyl)ethyl ester	9.992	487.22004	452.25135	Unknown	
Osmanthuside A	1.275	445.14833	446.15547	Unknown	
p-Anisic acid	5.121	151.04047	152.04764	Phenolics	[23,26,28,29]
Pantothenic Acid	2.325	218.10344	219.11059	Carbohydrate	[22]
Phe Gln Cys	10.04	395.14093	396.14923	Unknown	
Phe-Phe-OH	8.36	455.1035	420.13444	Unknown	
Phomopsin A	9.462	823.25172	788.28311	Unknown	
Pirenzepine	7.02	350.16201	351.16907	Flavonoids	[24]
Podolactone B	8.382	393.11983	394.12758	Unknown	
Polyethylene	9.984	243.12483	244.13204	Unknown	
Prasugrel	0.94	372.10699	373.11437	Unknown	
Procaterol	9.339	325.13113	290.16163	Unknown	
Prostaglandin M	6.886	327.1464	328.15356	Unknown	
Pumilaisoflavone B	9.54	463.17725	464.18226	Unknown	
p-Salicylic acid	9.691	137.0249	138.03214	Flavonoids	
Pseudomonine	7.478	329.12565	330.13297	Unknown	
Pymetrozine	4.305	216.08865	217.09607	Unknown	
Pyrimidifen	7.763	753.36276	377.18236	Unknown	
Quinic acid	1.733	191.05593	192.06314	Phenolics	[24]
Sandoricin	10.709	587.25136	588.25887	Unknown	
Schizonepetoside C	8.254	329.15925	330.16633	Unknown	
Scopolin	7.497	353.08966	354.09698	Unknown	
Scutellarein 5-glucuronide	9.323	461.07476	462.08215	Unknown	
Semilepidinoside A	8.2	371.10076	336.13199	Unknown	
Senkirkine	7.206	364.17768	365.18464	Unknown	
Septentriodine	9.546	735.32993	700.36121	Unknown	
Sesamex	8.316	297.1338	298.13951	Unknown	
Sulfometuron	7.517	349.06195	350.06935	Unknown	
Sudan Brown RR	10.168	523.23569	262.12103	Unknown	
Taraxacolide 1-O-b-D-glucopyranoside	8.53	855.40326	428.20581	Unknown	
Tetraneurin A	9.749	357.11227	322.14331	Pseudo guaianolides	[22]
Tetranor-PGEM	8.757	325.12836	326.13554	Unknown	
Tolbutamide	7.664	305.07242	270.10384	Unknown	
Torasemide	9.091	347.11884	348.12624	Flavonoids	[25]
Toyocamycin	0.844	290.09081	291.09822	Unknown	
Trans-trismethoxy Resveratrol-d4	1.263	309.12077	274.15123	Unknown	
Trimethylolpropane triacrylate	9.781	295.11991	296.12733	Unknown	
Trp Glu Leu	7.86	891.42472	446.21425	Unknown	
Trp Ser Pro	7.828	387.16782	388.17496	Unknown	
Trp Thr Ile	9.228	417.21434	418.22135	Unknown	
Tutin	9.327	293.10437	294.11161	Unknown	
Ustiloxin D	9.905	493.23059	494.23751	Unknown	
Val Trp Glu	7.589	431.194	432.20106	Unknown	
Vanilloloside	7.238	315.10961	316.117	Unknown	
Vinylacetylglycine	1.368	142.05147	143.0587	Unknown	

Note: *m*/*z* = mass number/charge number means mass-to-change ratio.

**Table 3:** Phytotoxic compounds of *P. hysterophorus* leaf with methanolic extracts through LC–MS analysis.

Sl No.	Compounds	Retention Time	*m*/*z*	Mass	Polarity	Synonyms	Chemical Formula	Chemical Structure	Biological Activity	References
1.	Caffeic acid	7.183	341.0894	342.09698	Negative	3-4-Dihydroxy cinnamic acid	C_9_H_8_O_4_	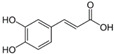	Antifungal, dermatitis, autotoxic, inhibitory effect to other plants	[22,23,24,25,26,27,28,29,30,31,32]
						3-(3,4-dihydroxy phenyl) acrylic acid				
2.	Ferulic acid	9.84	193.05129	194.05855	Negative	Trans-ferulic acid	C_10_H_10_O_4_	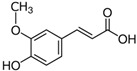		
						4-hydroxy-3-methoxy cinnamic acid				
						Coniferic acid				
						2 Propenoic acid, 3-(4-hydroxy-3-methoxy phenyl)				
3.	Vanillic acid	7.367	153.01983	154.02711	Negative	4-hydroxy-3-methoxybenzoic acid	C_8_H_8_O_4_	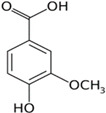		
						Benzoic acid, 4-hydroxy-3-methoxy				
4.	Quinic acid	12.116	181.12	180.1129	Positive	D-(-)-Quinic acid	C_7_H_12_O_6_	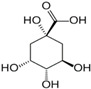		
						Chinic acid				
						Quinate				
						1,3,4,5-tetrahydroxy cyclohexanecarboxylic acid				
5.	Parthenin	10.004	263.1267	262.1194	Positive	10-alpha-H-Ambrosa-2,11(13)-1,6-beta di-hydroxy-4-oxo-,gamma –lactone	C_15_H_18_O_4_	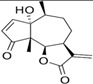		
						Grosshemin				
						Helenalin				
6.	Chlorogenic acid	8.09	300.183	282.1421	Positive	3, O -caffeoylquinic acid	C_16_H_18_O_9_	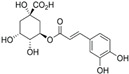		
						3-(3,4-dihydroxy cinnamoyl) quinic acid				
						3-caffeoylquinic acid				
						1,3,4,5-tetrahydroxy cyclohexanecarboxylic acid				
7.	p-Anisic acid	5.121	151.04047	152.04764	Negative	4-methoxy benzoic acid	C_8_H_8_O_3_	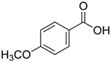		
						p-anisic acid				
						p-methoxybenzoic acid				

**Table 4:** Germination (%) of *D. sanguinalis* treated with selected phytochemicals.

Compounds	Concentration (μM)					
	0.00	100	200	400	800	1600
Caffeic acid	83.33 ± 3.33 aA (0)	71.11 ± 1.92 bB (14.66)	54.44 ± 1.92 cC (34.66)	45.55 ± 1.92 dB (45.33)	33.31 ± 3.30 eC (60.02)	0.00 fE (100.00)
Vanillic acid	83.33 ± 3.33 aA (0)	75.55 ± 1.92 bA (9.33)	69.99 ± 3.33 cA (16.00)	64.44 ± 1.92 dA (22.66)	58.88 ± 1.92 eA (29.34)	53.33 ± 3.33 fAB (36.00)
Ferulic acid	83.33 ± 3.33 aA (0)	73.33 ± 3.33 bAB (12.00)	66.66 ± 3.33 cAB (20.00)	61.11 ± 1.92 dA (26.66)	55.55 ± 1.92 eAB (33.33)	52.22 ± 1.92 eAB (37.33)
Chlorogenic acid	83.33 ± 3.33 aA (0)	73.33 ± 3.33 bAB (12.00)	67.77 ± 1.92 bcAB (18.67)	63.33 ± 3.33 cA (24.00)	53.33 ± 3.33 dB (36.00)	43.33 ± 3.33 eC (48.00)
Quinic acid	83.33 ± 3.33 aA (0)	72.22 ± 1.92 bAB (13.33)	66.66 ± 3.33 bcAB (20.00)	64.44 ± 5.09 cA (22.66)	59.99 ± 3.33 cA (28.00)	52.21 ± 5.09 dAB (37.34)
*p*-Anisic acid	83.33 ± 3.33 aA (0)	73.33 ± 3.33 bAB (12.00)	64.44 ± 5.09 cB (22.66)	61.10 ± 5.09 cdA (26.67)	59.99 ± 3.33 cdA (28.00)	56.66 ± 3.33 dA (32.00)
Parthenin	83.33 ± 3.33 aA (0)	74.44 ± 1.92 bAB (10.66)	64.44 ± 1.92 cB (22.66)	62.22 ± 1.92 cA (25.33)	53.33 ± 3.33 dB (36.00)	33.33 ± 3.33 eD (60.00)
Mixture (all compounds)	83.33 ± 3.33 aA (0)	72.22 ± 1.92 bAB (13.33)	68.88 ± 1.92 bAB (17.34)	63.33 ± 3.33 cA (24.00)	59.99 ± 3.33 cA (28.00)	48.88 ± 1.92 dB (41.34)
CV (%)	3.99	3.47	4.65	5.48	5.59	7.33
LSD (0.05)	5.76	4.40	5.26	5.76	5.25	5.39

Data are the means ± Standard Error. Means with the same small letters in the rows for each compound and the same capital letters within the concentrations (in columns) are not significantly different at *p* < 0.05. Figures in parentheses indicate the percent reduction in comparison to the control treatment.

**Table 5:** Root length (cm) of *D. sanguinalis* treated with selected phytochemicals.

Compounds	Concentration (μM)					
	0.00	100	200	400	800	1600
Caffeic acid	2.68 ± 0.03 aA (0)	1.46 ± 0.02 bG (45.52)	1.24 ± 0.02 cF (53.73)	1.12 ± 0.01 dF (58.20)	0.63 ± 0.02 eE (76.49)	0.00 fG (100.00)
Vanillic acid	2.68 ± 0.03 aA (0)	2.46 ± 0.02 bA (8.20)	2.36 ± 0.03 cA (11.94)	2.06 ± 0.03 dA (23.13)	1.65 ± 0.01 eA (38.43)	1.13 ± 0.02 fCD (57.83)
Ferulic acid	2.68 ± 0.03 aA (0)	1.88 ± 0.02 bC (29.85)	1.79 ± 0.01 cB (33.20)	1.66 ± 0.02 dB (38.05)	1.56 ± 0.01 eB (41.79)	1.42 ± 0.01 fA (47.01)
Chlorogenic acid	2.68 ± 0.03 aA (0)	1.78 ± 0.02 bDE (33.58)	1.68 ± 0.02 bcC (37.31)	1.55 ± 0.01 dD (42.16)	1.42 ± 0.02 eC (47.01)	1.23 ± 0.02 fB (54.10)
Quinic acid	2.68 ± 0.03 aA (0)	1.75 ± 0.01 bE (34.70)	1.60 ± 0.01 bcD (40.29)	1.51 ± 0.02 dD (43.65)	1.44 ± 0.02 eC (46.26)	0.87 ± 0.01 fE (67.53)
*p*-Anisic acid	2.68 ± 0.03 aA (0)	1.94 ± 0.02 bB (27.61)	1.70 ± 0.02 cC (36.56)	1.63 ± 0.02 dBC (39.17)	1.51 ± 0.02 eB (43.65)	1.10 ± 0.01 fD (58.95)
Parthenin	2.68 ± 0.03 aA (0)	1.59 ± 0.03 bF (40.67)	1.40 ± 0.03 cE (47.76)	1.25 ± 0.02 dE (53.35)	1.07 ± 0.07 eD (60.07)	0.56 ± 0.01 fF (79.10)
Mixture (all compounds)	2.68 ± 0.03 aA (0)	1.82 ± 0.02 bD (32.08)	1.68 ± 0.03 cC (37.31)	1.61 ± 0.02 dC (39.92)	1.42 ± 0.01 eC (47.01)	1.14 ± 0.02 fC (57.46)
CV (%)	1.13	1.27	1.49	1.46	2.50	1.78
LSD (0.05)	0.05	0.04	0.04	0.03	0.05	0.02

Data are the means ± Standard Error. Means with the same small letters in the rows for each compound and the same capital letters within the concentrations (in columns) are not significantly different at *p* < 0.05. Figures in parentheses indicate the percent reduction in comparison to the control treatment.

**Table 6:** Shoot length (cm) of *D. sanguinalis* treated with detected allelochemicals.

Compounds	Concentration (μM)					
	0.00	100	200	400	800	1600
Caffeic acid	2.98 ± 0.03 aA (0)	2.58 ± 0.02 bC (13.42)	2.12 ± 0.02 cF (28.85)	1.56 ± 0.02 dF (47.65)	0.86 ± 0.01 eF (71.14)	0.00 fE (100)
Vanillic acid	2.98 ± 0.03 aA (0)	2.89 ± 0.04 bA (3.02)	2.68 ± 0.02 cB (10.06)	2.41 ± 0.02 dC (19.12)	1.82 ± 0.03 eDE (38.92)	1.41 ± 0.02 fC (52.68)
Ferulic acid	2.98 ± 0.03 aA (0)	2.73 ± 0.03 bB (8.38)	2.64 ± 0.02 cC (11.40)	2.52 ± 0.01 dB (15.43)	2.02 ± 0.03 eC (32.21)	1.67 ± 0.01 fA (43.95)
Chlorogenic acid	2.98 ± 0.03 aA (0)	2.73 ± 0.02 bB (8.38)	2.66 ± 0.01 cBC (10.73)	2.43 ± 0.02 dC (18.45)	2.22 ± 0.02 eB (25.50)	1.69 ± 0.02 fA (43.28)
Quinic acid	2.98 ± 0.03 aA (0)	2.62 ± 0.02 bC (12.08)	2.33 ± 0.02 cE (21.81)	2.19 ± 0.02 dE (26.51)	1.83 ± 0.02 eD (38.59)	1.63 ± 0.02 fB (45.30)
*p*-Anisic acid	2.98 ± 0.03 aA (0)	2.85 ± 0.02 bA (4.36)	2.76 ± 0.02 cA (7.38)	2.70 ± 0.01 dA (9.39)	2.53 ± 0.02 eA (15.10)	1.67 ± 0.02 fA (43.95)
Parthenin	2.98 ± 0.03 aA (0)	2.61 ± 0.02 bC (12.41)	2.31 ± 0.03 cE (22.48)	2.21 ± 0.01 dE (25.83)	1.81 ± 0.02 eDE (39.26)	1.11 ± 0.01 fD (62.75)
Mixture (all compounds)	2.98 ± 0.03 aA (0)	2.71 ± 0.02 bB (9.06)	2.53 ± 0.02 cD (15.10)	2.32 ± 0.02 dD (22.14)	1.78 ± 0.02 eE (40.26)	1.43 ± 0.03 fC (52.01)
CV (%)	1.17	0.95	0.92	0.94	1.27	1.58
LSD (0.05)	0.06	0.04	0.03	0.03	0.04	0.03

Data are the means ± Standard Error. Means with the same small letters in the rows for each compound and the same capital letters within the concentrations (in columns) are not significantly different at *p* < 0.05. Figures in parentheses indicate the percent reduction in comparison to the control treatment.

**Table 7:** Inhibitory effect (EC_50_) of phytotoxic compounds, the sensitivity of examined initial growth parameters of *D. sanguinalis*.

Allelopathic Compounds	EC_g50_	EC_r50_	EC_s50_	Rank
	Values in μM (Lower–Upper)			
Caffeic acid	379.1(124.7–1054.7)	168.5	361.9 (66.8–1471.1)	909.5
Vanillic acid	4228.9(2024.1–23321.2)	1251.0	1349.7	6829.6
Ferulic acid	3893.5(1797.5–27099.9)	2650.6	2334.7	8878.8
Chlorogenic acid	1927.9(1217.9–4441.5)	1060.6	2792.6	5781.1
Quinic acid	6081.9(2284.1–112731.5)	562.3	2003.2	8647.4
*p*-Anisic acid	10972.3(2881.5–3224192.2)	956.3	2917.2	14845.8
Parthenin	1234.1(895.3–2009.4)	245.2	1090.1	2569.4
Mixture (all compounds)	3880.2(1843.9–23076.2)	916.7	1442.3	6239.2
Rank	32,597.9	7811.2	14,291.7	54,700.8

EC_g50_, EC_r50_ and EC_h50_ are the concentrations of compounds that inhibit 50% germination, root, and hypocotyl, respectively.

**Table 8:** Germination (%) of *E. indica* treated with the phytochemicals.

Compounds	Concentration (μM)					
	0.00	100	200	400	800	1600
Caffeic acid	94.44 ± 1.92 aA (0)	73.33 ± 3.33 bC (22.35)	55.55 ± 1.92 cC (41.17)	47.77 ± 1.92 dE (49.41)	0.00 eE (100)	0.00eE (100)
Vanillic acid	94.44 ± 1.92 aA (0)	92.21 ± 5.09 aA (2.36)	79.99 ± 3.33 bB (15.30)	71.11 ± 1.92 cCD (24.70)	63.33 ± 3.33 dC (32.94)	47.77 ± 1.92 eB (49.41)
Ferulic acid	94.44 ± 1.92 aA (0)	89.99 ± 3.33 bA (4.71)	81.11 ± 1.92 cB (14.11)	76.66 ± 3.33 dB (18.82)	71.11 ± 1.92 eB (24.70)	51.11 ± 1.92 fB (45.88)
Chlorogenic acid	94.44 ± 1.92 aA (0)	79.99 ± 3.33 bB (15.30)	78.88 ± 1.92 bB (16.47)	67.77 ± 1.92 cD (28.24)	53.33 ± 3.33 dD (43.53)	33.33 ± 3.33 eC (64.70)
Quinic acid	94.44 ± 1.92 aA (0)	92.22 ± 1.92 aA (2.35)	91.11 ± 1.92 aA (3.52)	84.44 ± 1.92 bA (10.58)	71.11 ± 1.92 cB (24.70)	49.99 ± 3.33 dB (47.06)
*p*-Anisic acid	94.44 ± 1.92 aA (0)	93.33 ± 3.84 aA (2.35)	92.22 ± 3.33 aA (1.17)	83.33 ± 3.33 bA (11.76)	83.33 ± 3.33 bA (11.76)	79.99 ± 3.33 bA (15.30)
Parthenin	94.44 ± 1.92 aA (0)	87.77 ± 5.09 bA (7.06)	79.99 ± 3.33 cB (15.30)	71.11 ± 1.92 dCD (24.70)	53.33 ± 3.33 eD (43.53)	21.11 ± 1.92 dD (77.64)
Mixture (all compounds)	94.44 ± 1.92 aA (0)	79.99 ± 3.33 bB (15.30)	76.66 ± 3.33 bcB (18.82)	73.33 ± 3.33 cdBC (22.35)	68.88 ± 1.92 dB (27.06)	49.99 ± 3.33 eB (47.06)
CV (%)	2.03	4.40	3.42	3.53	4.53	6.32
LSD (0.05)	3.32	6.55	4.71	4.40	4.55	4.56

Data are the means ± Standard Error. Means with the same small letters in the rows for each compound and the capital letter within the concentrations (in columns) are not significantly different at *p* < 0.05. Figures in parentheses indicate the percent reduction in comparison to the control treatment.

**Table 9:** Root length (cm) of *E. indica* treated with the phytochemicals.

Compounds	Concentration (μM)					
	0.00	100	200	400	800	1600
Caffeic acid	2.74 ± 0.035 aA (0)	1.49 ± 0.015 bG (45.62)	1.28 ± 0.02 CG (53.28)	0.403 ± 0.005 dG (85.29)	0.00 eF (100)	0.00eG (100)
Vanillic acid	2.74 ± 0.035 aA (0)	2.54 ± 0.020 bA (7.29)	2.45 ± 0.025 cA (10.58)	2.33 ± 0.025 dA (14.96)	1.55 ± 0.010 eB (43.43)	0.97 ± 0.010 fD (64.59)
Ferulic acid	2.74 ± 0.035 aA (0)	1.93 ± 0.025 bC (29.56)	1.90 ± 0.015 bB (30.65)	1.79 ± 0.01 CB (34.67)	1.77 ± 0.01 CA (35.40)	1.40 ± 0.005 dA (48.90)
Chlorogenic acid	2.74 ± 0.035 aA (0)	1.83 ± 0.032 bDE (33.57)	1.82 ± 0.020 bC (33.21)	1.74 ± 0.015 cC (36.49)	1.50 ± 0.015 dC (45.25)	1.40 ± 0.020 eA (48.90)
Quinic acid	2.74 ± 0.035 aA (0)	1.80 ± 0.025 bE (34.30)	1.79 ± 0.020 bCD (34.67)	1.60 ± 0.015 cE (41.60)	1.55 ± 0.010 dB (43.43)	0.67 ± 0.010 eE (75.54)
*p*-Anisic acid	2.74 ± 0.035 aA (0)	2.02 ± 0.025 bB (26.27)	1.73 ± 0.025 cE (36.86)	1.70 ± 0.025 cD (37.95)	1.45 ± 0.015 dD (47.08)	1.10 ± 0.011 eC (59.85)
Parthenin	2.74 ± 0.035 aA (0)	1.62 ± 0.025 bF (40.87)	1.60 ± 0.026 bF (41.60)	0.96 ± 0.015 cF (65.32)	0.95 ± 0.01 CE (64.96)	0.56 ± 0.015 dF (79.56)
Mixture (all compounds)	2.74 ± 0.035 aA (0)	1.86 ± 0.025 bD (32.11)	1.79 ± 0.026 cD (34.67)	1.68 ± 0.030 dD (38.68)	1.51 ± 0.020 eC (44.89)	1.16 ± 0.020 fB (57.66)
CV (%)	1.28	1.30	1.26	1.26	0.98	1.48
LSD (0.05)	0.06	0.04	0.03	0.03	0.02	0.02

Data are the means ± Standard Error. Means with the same small letters in the rows for each compound and the same capital letters within the concentrations (in columns) are not significantly different at *p* < 0.05. Figures in parentheses indicate the percent reduction in comparison to the control treatment.

**Table 10:** Shoot length (cm) of *E. indica* treated with the phytochemicals.

Compounds	Concentration (μM)					
	0.00	100	200	400	800	1600
Caffeic acid	3.09 ± 0.068 aA (0)	2.71 ± 0.011 bE (12.29)	2.23 ± 0.03 CF (27.83)	1.66 ± 0.015 dH (46.27)	0.00 eG (100)	0.00 eF (100)
Vanillic acid	3.09 ± 0.068 aA (0)	3.03 ± 0.020 bA (1.94)	2.88 ± 0.02 CB (6.79)	2.09 ± 0.025 dG (32.36)	1.77 ± 0.010 eF (42.71)	1.42 ± 0.015 fD (54.04)
Ferulic acid	3.09 ± 0.068 aA (0)	2.86 ± 0.010 bBC (7.44)	2.73 ± 0.025 cC (11.65)	2.72 ± 0.023 cB (11.97)	2.04 ± 0.02 C (33.98)	1.69 ± 0.010 eA (45.30)
Chlorogenic acid	3.09 ± 0.068 aA (0)	2.86 ± 0.015 bB (7.44)	2.76 ± 0.025 cC (10.67)	2.44 ± 0.020 dD (21.03)	2.24 ± 0.025 eB (27.50)	1.67 ± 0.010 fA (45.95)
Quinic acid	3.09 ± 0.068 aA (0)	2.75 ± 0.015 bD (11.00)	2.42 ± 0.015 cE (21.68)	2.36 ± 0.010 dF (23.62)	1.94 ± 0.015 eD (37.21)	1.61 ± 0.020 fB (47.89)
*p*-Anisic acid	3.09 ± 0.068 aA (0)	3.01 ± 0.035 bA (2.58)	2.98 ± 0.025 bA (3.55)	2.89 ± 0.011 cA (6.47)	2.75 ± 0.010 dA (11.00)	1.67 ± 0.011 eA (45.95)
Parthenin	3.09 ± 0.068 aA (0)	2.75 ± 0.020 bD (11.00)	2.43 ± 0.02 CE (21.35)	2.39 ± 0.015 cE (22.65)	1.86 ± 0.020 dE (39.80)	0.20 ± 0.010 eE (93.52)
Mixture (all compounds)	3.09 ± 0.068 aA (0)	2.83 ± 0.020 bC (8.41)	2.65 ± 0.02 CD (14.23)	2.51 ± 0.025 dC (18.77)	1.86 ± 0.025 eE (39.80)	1.46 ± 0.025 fC (52.75)
CV (%)	2.19	0.73	0.89	0.80	0.98	1.19
LSD (0.05)	0.11	0.03	0.04	0.03	0.03	0.02

Data are the means ± Standard Error. Means with the same small letters in the rows for each compound and the same capital letters within the concentrations (in columns) are not significantly different at *p* < 0.05. Figures in parentheses indicate the percent reduction in comparison to the control treatment.

**Table 11:** Inhibitory effect of phytotoxic compounds, the sensitivity of examined initial growth parameters of *E. indica*.

Allelopathic Compounds	EC_g50_	EC_r50_	EC_s50_	Rank
	Values in μM (Lower–Upper)			
Caffeic acid	246.18 (30.74–672.05)	138.00	300.52 (96.31–764.42)	684.7
Vanillic acid	1558.74 (1158.61–2415.20)	1074.88	1125.16	3758.78
Ferulic acid	2298.80 (1536.33–4482.93)	3549.40	2121.82	7970.02
Chlorogenic acid	976.58 (755.64–1384.74)	2149.42	2221.36	5347.36
Quinic acid	1870.23 (1438.35–2748.53)	545.58	1865.92	4281.73
*p*-Anisic acid	16271.87 (5369.83–315463.09)	849.02	2432.36	19553.25
Parthenin	795.38 (670.38–973.10)	221.41	620.87	1637.66
Mixture (all compounds)	3131.83 (1662.03–s12079.69)	1029.80	1452.17	5613.8
Rank	27,149.61	9557.51	12,140.18	48,847.3

EC_g50_, EC_r50_, and EC_h50_ are the concentrations of compounds that inhibit a 50% germination, root growth, and hypocotyl elongation, respectively.

## Data Availability

The data presented in this study are available in this article.

## References

[1] Kwinda M. (2021). Weed Profiling Fields of Herbicide Tolerant Maize in the Mthatha Region, Eastern Cape Province. Ph.D. Thesis.

[2] Chuah T.S., Lim W.K. (2021). Combination Ratio Affects Synergistic Activity of Oil Palm Frond Residue and S-Metolachlor on Goosegrass (*Eleusine indica*). Pakistan J. Bot..

[3] Chuah T.S., Lim W.K. (2015). Assessment of Phytotoxic Potential of Oil Palm Leaflet, Rachis and Frond Extracts and Powders on Goosegrass (*Eleusine indica* (L.) Gaertn) Germination, Emergence and Seedling Growth. Malaysian Appl. Biol..

[4] Chauhan B.S., Johnson D.E. (2008). Germination Ecology of Goosegrass (*Eleusine indica*): An Important Grass Weed of Rainfed Rice. Weed Sci..

[5] Shrestha A., Anwar M.P., Islam A., Gurung T., Dhakal S., Tanveer A., Javaid M.M., Nadeem M., Ikram N.A. (2021). Weed Science as a New Discipline and Its Status in Some South Asian Universities and Colleges: Examples from Bangladesh, Bhutan, Nepal and Pakistan. CAB Rev..

[6] Sunohara Y., Shirai S., Wongkantrakorn N., Matsumoto H. (2010). Sensitivity and Physiological Responses of *Eleusine indica* and *Digitaria adscendens* to Herbicide Quinclorac and 2,4-D. Environ. Exp. Bot..

[7] Khaket T.P., Aggarwal H., Jodha D., Dhanda S., Singh J. (2015). *Parthenium hysterophorus* in current scenario: A toxic weed with industrial, agricultural and medicinal applications. J. Plant Sci..

[8] Scavo A., Mauromicale G. (2021). Crop Allelopathy for Sustainable Weed Management in Agroecosystems: Knowing the Present with a View to the Future. Agronomy.

[9] Motmainna M., Juraimi A.S., Uddin M.K., Asib N.B., Islam A.K.M.M., Hasan M. (2021). Bioherbicidal Properties of *Parthenium hysterophorus*, *Cleome rutidosperma* and *Borreria alata* Extracts on Selected Crop and Weed Species. Agronomy.

[10] Ambika S.R. (2013). Multifaceted Attributes of Allelochemicals and Mechanism of Allelopathy. Allelopathy.

[11] Aslani F., Juraimi A.S., Ahmad-Hamdani M.S., Hashemi F.S.G., Alam M.A., Hakim M.A., Uddin M.K. (2016). Effects of *Tinospora tuberculata* Leaf Methanol Extract on Seedling Growth of Rice and Associated Weed Species in Hydroponic Culture. J. Integr. Agric..

[12] Hao W., Ren L., Ran W., Shen Q. (2010). Allelopathic Effects of Root Exudates from Watermelon and Rice Plants on *Fusarium oxysporum* f. Sp. Niveum. Plant Soil.

[13] Belz R.G., Reinhardt C.F., Foxcroft L.C., Hurle K., Singh I. (2007). Residue Allelopathy in *Parthenium hysterophorus* L.-Does Parthenin Play a Leading Role?. Crop. Prot..

[14] Hasan M., Ahmad-Hamdani M.S., Rosli A.M., Hamdan H. (2021). Bioherbicides: An eco-friendly tool for sustainable weed management. Plants.

[15] Holt J.S., Welles S.R., Silvera K., Heap I.M., Heredia S.M., Martinez-Berdeja A., Palenscar K.T., Sweet L.C., Ellstrand N.C. (2013). Taxonomic and Life History Bias in Herbicide Resistant Weeds: Implications for Deployment of Resistant Crops. PLoS ONE.

[16] Dilipkumar M., Chuah T.S., Goh S.S., Sahid I. (2020). Weed Management Issues, Challenges, and Opportunities in Malaysia. Crop. Prot..

[17] Saini A., Aggarwal N.K., Sharma A., Kaur M., Yadav A. (2014). Utility Potential of Parthenium hysterophorus for Its Strategic Management. Adv. Agric..

[18] Hossen K., Ozaki K., Teruya T., Kato-noguchi H. (2021). Three Active Phytotoxic Compounds from the Leaves of *Albizia richardiana* (Voigt.) King and Prain for the Development of Bioherbicides to Control Weeds. Cells.

[19] De Mastro G., El Mahdi J., Ruta C. (2021). Bioherbicidal Potential of the Essential Oils from *Mediterranean lamiaceae* for Weed Control in Organic Farming. Plants.

[20] Sharma A., Shukla A., Attri K., Kumar M., Kumar P., Suttee A., Singh G., Barnwal R.P., Singla N. (2020). Global Trends in Pesticides: A Looming Threat and Viable Alternatives. Ecotoxicol. Environ. Saf..

[21] Thien B.N., Ba V.N., Man M.T., Loan T.T.H. (2021). Analysis of the Soil to Food Crops Transfer Factor and Risk Assessment of Multi-Elements at the Suburban Area of Ho Chi Minh City, Vietnam Using Instrumental Neutron Activation Analysis (INAA). J. Environ. Manag..

[22] Patel S. (2011). Harmful and beneficial aspects of Parthenium hysterophorus: An update. 3 Biotech.

[23] Xie G., Zhou J., Yan X. (2011). Encyclopedia of Traditional Chinese Medicines: Molecular Structures, Pharmacological Activities, Natural Sources and Applications.

[24] Pareek A., Suthar M., Rathore G.S., Bansal V. (2011). Feverfew (*Tanacetum parthenium* L.): A Systematic Review. Pharmacogn. Rev..

[25] Iqbal J., Khan A.A., Aziz T., Ali W., Ahmad S., Rahman S.U., Iqbal Z., Dablool A.S., Alruways M.W., Almalki A.A. (2022). Phytochemical Investigation, Antioxidant Properties and In Vivo Evaluation of the Toxic Effects of Parthenium hysterophorus. Molecules.

[26] NTSYS-pc N.T., Taxonomy N. (2005). Multivariate Analysis System.

[27] Tarinezhad A., Sabouri A., Mohammadi S.A. Statistical Software NTSYS PC Application in Plant Breeding. The 7th Conference of Iran Statistics. Allame Tabatabaei University, Tehran, Iran, September 2005. http://irstat.ir/files/site1/files/conference/7thconference_(English).pdf.

[28] Parsons W.T., Parsons W.T., Cuthbertson E.G. (2001). Noxious Weeds of Australia.

[29] Petersen J., Belz R., Walker F., Hurle K. (2001). Weed Suppression by Release of Isothiocyanates from Turnip-rape Mulch. Agron. J..

[30] Bezuneh T.T. (2015). Phytochemistry and antimicrobial activity of *Parthenium hysterophorus* L.: A review. Sci. J. Anal. Chem..

[31] Pandey R.A., Gole A.R., Sankpal R.V., Jadav P.V., Waghmode M.S., Patil N.N. (2019). Bioactive Potential of *Parthenium hysterophorus* and Cytotoxicity Assay of Parthenin. Int. J. Pharm. Biol. Sci..

[32] Marwat S.K., Fazal-ur-Rehman, Khan I.U. (2015). Ethnobotanical Importance and Phytochemical Constituents of Parthenium Weed (*Parthenium hysterophorus* L.)—A Review. Plant Sci. Today.

[33] Roy D.C., Shaik M. (2013). Journal of Medicinal Plants Studies Toxicology, Phytochemistry, Bioactive Compounds and Pharmacology of *Parthenium hysterophorus*. J. Med. Plants Stud..

[34] Verdeguer M., Blázquez M.A., Boira H. (2009). Phytotoxic Effects of *Lantana camara*, *Eucalyptus camaldulensis* and *Eriocephalus africanus* Essential Oils in Weeds of Mediterranean Summer Crops. Biochem. Syst. Ecol..

[35] Javaid A., Anjum T. (2006). Control of *Parthenium hysterophorus* L., by Aqueous Extracts of Allelopathic Grasses. Pakistan J. Bot..

[36] Verma A.K., Maurya S.K., Kumar A., Barik M., Yadav V., Umar B., Lawal M., Usman Z.A., Adam M.A., Awal B. (2020). Inhibition of Multidrug Resistance Property of Candida Albicans by Natural Compounds of *Parthenium hysterophorus* L. An In-Silico Approach. J. Pharmacogn. Phytochem..

[37] Asaduzzaman M., Asao T. (2020). Autotoxicity in Strawberry under Recycled Hydroponics and Its Mitigation Methods. Hortic. J..

[38] Batish D.R., Singh H.P., Kaur S., Kohli R.K., Yadav S.S. (2008). Caffeic Acid Affects Early Growth, and Morphogenetic Response of Hypocotyl Cuttings of Mung Bean (*Phaseolus aureus*). J. Plant Physiol..

[39] Kaur S., Singh H.P., Mittal S., Batish D.R., Kohli R.K. (2010). Phytotoxic Effects of Volatile Oil from *Artemisia scoparia* against Weeds and Its Possible Use as a Bioherbicide. Ind. Crops Prod..

[40] Sharma A., Singh H.P., Batish D.R., Kohli R.K. (2019). Chemical Profiling, Cytotoxicity and Phytotoxicity of Foliar Volatiles of *Hyptis suaveolens*. Ecotoxicol. Environ. Saf..

[41] Reigosa M.J., Pazos-Malvido E. (2007). Phytotoxic Effects of 21 Plant Secondary Metabolites on *Arabidopsis thaliana* Germination and Root Growth. J. Chem. Ecol..

[42] Bajwa A.A., Weston P.A., Gurusinghe S., Latif S., Adkins S.W., Weston L.A. (2020). Toxic Potential and Metabolic Profiling of Two Australian Biotypes of the Invasive Plant Parthenium Weed (*Parthenium hysterophorus* L.). Toxins.

[43] Guo Y., Kim K.-U., Yoder J.I., Shin D. (2011). Parasitic Plants as a New Target Plant for Screening Rice Allelopathic Potential. J. Life Sci..

[44] Rasouli H., Farzaei M.H., Mansouri K., Mohammadzadeh S., Khodarahmi R. (2016). Plant Cell Cancer: May Natural Phenolic Compounds Prevent Onset and Development of Plant Cell Malignancy? A Literature Review. Molecules.

[45] Macías F.A., Mejías F.J.R., Molinillo J.M.G. (2019). Recent Advances in Allelopathy for Weed Control: From Knowledge to Applications. Pest Manag. Sci..

[46] Latif S., Chiapusio G., Weston L.A., Becard G. (2017). Allelopathy and the Role of Allelochemicals in Plant Defence. How Plants Communicate with Their Biotic Environment.

[47] Aslam F., Khaliq A., Matloob A., Tanveer A., Hussain S., Zahir Z.A. (2017). Allelopathy in Agro-Ecosystems: A Critical Review of Wheat Allelopathy-Concepts and Implications. Chemoecology.

[48] Amarowicz R., Cwalina-Ambroziak B., Janiak M.A., Bogucka B. (2020). Effect of N Fertilization on the Content of Phenolic Compounds in Jerusalem Artichoke (*Helianthus tuberosus* L.) Tubers and Their Antioxidant Capacity. Agronomy.

[49] Braga T.M., Rocha L., Chung T.Y., Oliveira R.F., Pinho C., Oliveira A.I., Morgado J., Cruz A. (2020). Biological Activities of Gedunin—A Limonoid from the Meliaceae Family. Molecules.

[50] Abbas T., Tanveer A., Khaliq A., Safdar M.E., Nadeem M.A. (2014). Allelopathic Effects of Aquatic Weeds on Germination and Seedling Growth of Wheat. Herbologia.

[51] Scognamiglio M., Esposito A., D’Abrosca B., Pacifico S., Fiumano V., Tsafantakis N., Monaco P., Fiorentino A. (2012). Isolation, Distribution and Allelopathic Effect of Caffeic Acid Derivatives from *Bellis perennis* L. Biochem. Syst. Ecol..

[52] Krumsri R., Iwasaki A., Suenaga K., Kato-Noguchi H. (2022). Assessment of Allelopathic Potential of *Senna garrettiana* Leaves and Identification of Potent Phytotoxic Substances. Agronomy.

[53] Scognamiglio M., D’Abrosca B., Esposito A., Pacifico S., Monaco P., Fiorentino A. (2013). Plant Growth Inhibitors: Allelopathic Role or Phytotoxic Effects? Focus on Mediterranean Biomes. Phytochem. Rev..

[54] Safdar M.E., Aslam A., Qamar R., Ali A., Javaid M.M., Hayyat M.S., Raza A. (2021). Allelopathic Effect of Prickly Chaff Flower (*Achyranthes aspera* L.) Used as a Tool for Managing Noxious Weeds. Asian J. Agric. Biol..

[55] Ahn J.K., Chung I.M. (2000). Allelopathic Potential of Rice Hulls on Germination and Seedling Growth of Barnyardgrass. Agron. J..

[56] Salam M.A., Kato-Noguchi H. (2010). Allelopathic potential of methanol extract of Bangladesh rice seedlings. Asian J. Crop Sci..

[57] Mao R., Shabbir A., Adkins S. (2021). *Parthenium hysterophorus*: A Tale of Global Invasion over Two Centuries, Spread and Prevention Measures. J. Environ. Manag..

[58] Schymanski E.L., Jeon J., Gulde R., Fenner K., Ruff M., Singer H.P., Hollender J. (2014). Identifying Small Molecules via High Resolution Mass Spectrometry: Communicating Confidence. Environ. Sci. Technol..

[59] Aslani F., Juraimi A.S., Ahmad-Hamdani M.S., Omar D., Alam M.A., Hashemi F.S.G., Hakim M.A., Uddin M.K. (2014). Allelopathic Effect of Methanol Extracts from Tinospora Tuberculata on Selected Crops and Rice Weeds. Acta Agric. Scand. Sect. B Soil Plant Sci..

[60] Mirmostafaee S., Azizi M., Fujii Y. (2020). Study of Allelopathic Interaction of Essential Oils from Medicinal and Aromatic Plants on Seed Germination and Seedling Growth of Lettuce. Agronomy.

